# Detection of cold pain, cold allodynia and cold hyperalgesia in freely behaving rats

**DOI:** 10.1186/1744-8069-1-36

**Published:** 2005-12-14

**Authors:** Andrew J Allchorne, Daniel C Broom, Clifford J Woolf

**Affiliations:** 1 Neural Plasticity Research Group, Department of Anesthesia & Critical Care, Massachusetts General Hospital & Harvard Medical School, 13th Street, Building 149 (#4309), Charlestown, MA 02129, USA; 2 Neurogen Corporation, 35 NE Industrial Rd., Branford, CT 06405, USA

## Abstract

**Background:**

Pain is elicited by cold, and a major feature of many neuropathic pain states is that normally innocuous cool stimuli begin to produce pain (cold allodynia). To expand our understanding of cold induced pain states we have studied cold pain behaviors over a range of temperatures in several animal models of chronic pain.

**Results:**

We demonstrate that a Peltier-cooled cold plate with ± 1°C sensitivity enables quantitative measurement of a detection withdrawal response to cold stimuli in unrestrained rats. In naïve rats the threshold for eliciting cold pain behavior is 5°C. The withdrawal threshold for cold allodynia is 15°C in both the spared nerve injury and spinal nerve ligation models of neuropathic pain. Cold hyperalgesia is present in the spared nerve injury model animals, manifesting as a reduced latency of withdrawal response threshold at temperatures that elicit cold pain in naïve rats. We also show that following the peripheral inflammation produced by intraplantar injection of complete Freund's adjuvant, a hypersensitivity to cold occurs.

**Conclusion:**

The peltier-cooled provides an effective means of assaying cold sensitivity in unrestrained rats. Behavioral testing of cold allodynia, hyperalgesia and pain will greatly facilitate the study of the neurobiological mechanisms involved in cold/cool sensations and enable measurement of the efficacy of pharmacological treatments to reduce these symptoms.

## Background

Neuropathic pain patients exhibit a wide range of symptoms including spontaneous and stimulus-evoked pain [1]. The latter include tactile allodynia (pain caused by a normally innocuous stimulus), pinprick hyperalgesia (heightened sensitivity to a painful stimulus), altered sensitivity to heat, cold hyperalgesia (heightened sensitivity to a painful cold stimulus) and cold allodynia (pain caused by a normally innocuous cold stimulus). Cold hyperalgesia is present in 9% of patients with varying neuropathies [2] and 23% of patients with post stroke central pain (CPSP) exhibited cold allodynia [3]. Many rodent models of neuropathic pain have been developed and characterized using standardized limb withdrawal measures as a surrogate for the sensory threshold or responsiveness to innocuous or noxious stimuli, such as; von Frey hairs and sensitivity to brush for mechanical allodynia, pin prick for mechanical hyperalgesia, hot plate and radiant heat for heat pain. A number of techniques have been employed for studying cold pain and cold allodynia using reflex responses (lifting of the hindpaw) or behavioral escape endpoints. These methods include: The application of a droplet of acetone [4,5] or ethyl chloride spray,[6] allowing the tester to cool, by evaporation, a small area of the animal's paw and measure hindpaw elevation as an index of pain-related behavior. However, the ambient temperature and the body temperature of the animal will affect the rate of evaporation of the liquid and therefore the temperature to which the skin is cooled, which is very difficult to determine accurately. One cannot be sure that the liquid elicits no chemical, olfactory or mechanical stimulus that may, independent of the temperature, elicit a flexion reflex. Dipping the foot of a restrained animal into a cooled water bath [7-9] causes a high level of stress, which may have nociceptive or anti-nociceptive effects that will confound the interpretation of any behavioral reflex response [10,11]. The use of an ice cooled metal plate [4] or shallow iced water bath [12] allows testing in unrestrained animals, but only at one temperature (4°C). Jasmin and colleagues [23] demonstrated the use of a liquid cooled cold plate with a testing temperature range above 0°C to measure the number of paw lifts over a four minute testing period rather than the latency to withdrawal. An alternative approach is the escape test paradigm, whereby a rat can preferentially move from a cold plate to one of neutral temperature [13]. The advantage here is that this is not a reflex, but a disadvantage is that this is a low throughput test.

Given the prevalence of cold sensitivity in neuropathic pain patients and the recent cloning of the cold-sensitive transient receptor potential (TRP) channels TRPM8 and TRPA1 [14-16] we sought to design a device which will allow the measurement of withdrawal latency in unrestrained rats over a range of accurately determined temperatures. This apparatus utilizes the Peltier effect, the creation of a heat differential by an electric voltage, allowing the surface temperature of a metal plate to be controlled.

In this study we assessed the responses of naïve rats over a temperature range of -5°C to 25°C to determine the threshold for cold pain. We also examined the cold temperature sensitivity of two rodent models of neuropathic pain, the spared nerve injury (SNI) [17] and the spinal nerve ligation (SNL) [18], both of which show increased cold sensitivity using the acetone test. Finally, we show changes in cold sensitivity following complete Freunds Adjuvant (CFA) inflammation of the hind paw.

## Results

### Temperature effect in naïve rats

**Figure 1:**
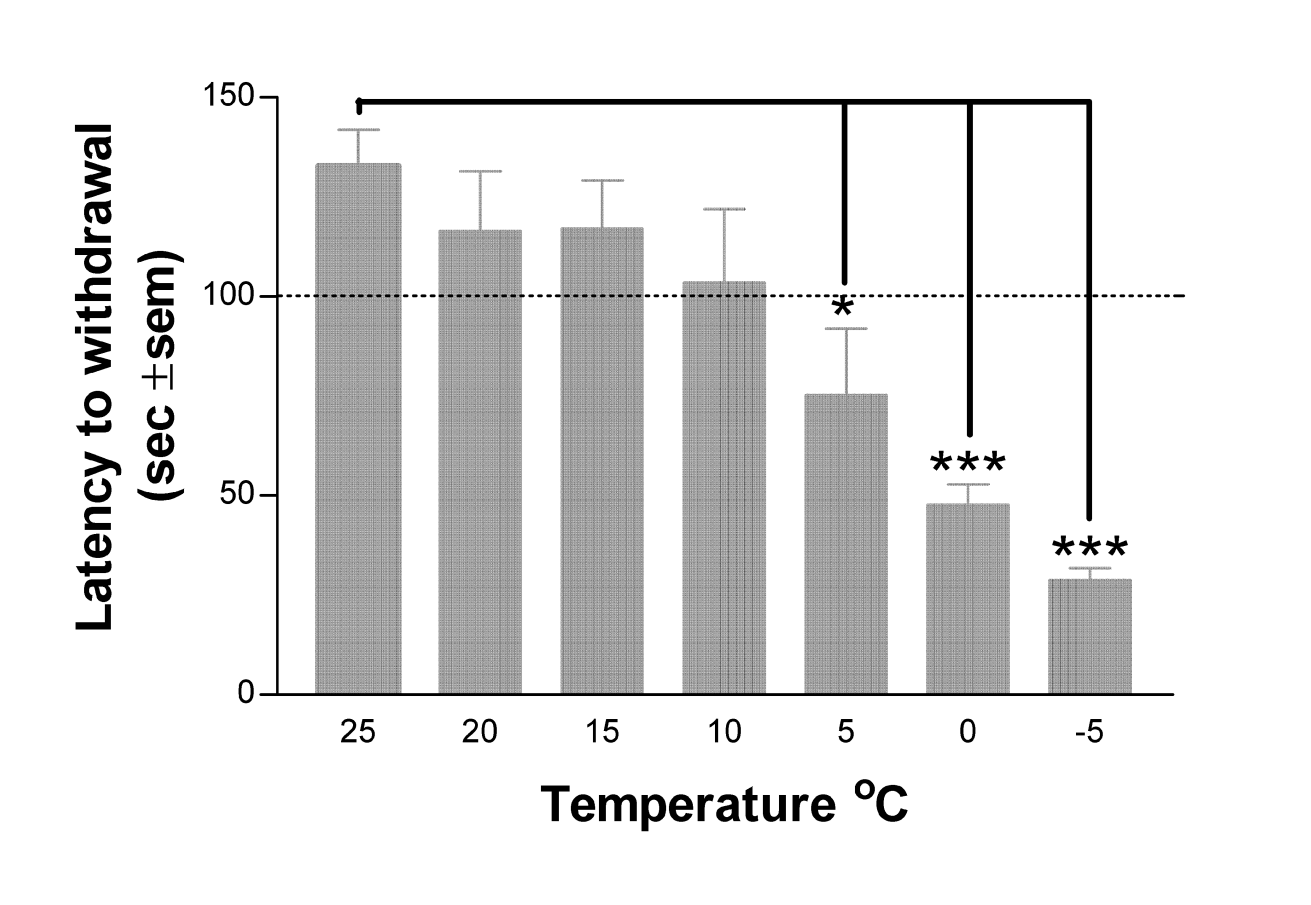
Cold pain threshold in naïve rats (n = 6). The mean latency for withdrawal ± S.E.M became significantly different from the baseline at surface temperatures of 5°C and below. Temperatures of 10°C and above can therefore be considered innocuous.

Naïve rats were tested over a temperature range of -5°C to 25°C at 5° intervals (Fig 1). The latency to withdrawal becomes shorter as the testing temperature is reduced and is significantly different from baseline (25°C) at temperatures of 5°C (p < 0.05) and below (0°C and -5°C p < 0.001). We conclude, therefore, that the threshold range for eliciting cold pain in naïve adult male Sprague-Dawley rats is between 5 and 9°C.

### Temperature effect in neuropathic pain models

**Figure 2:**
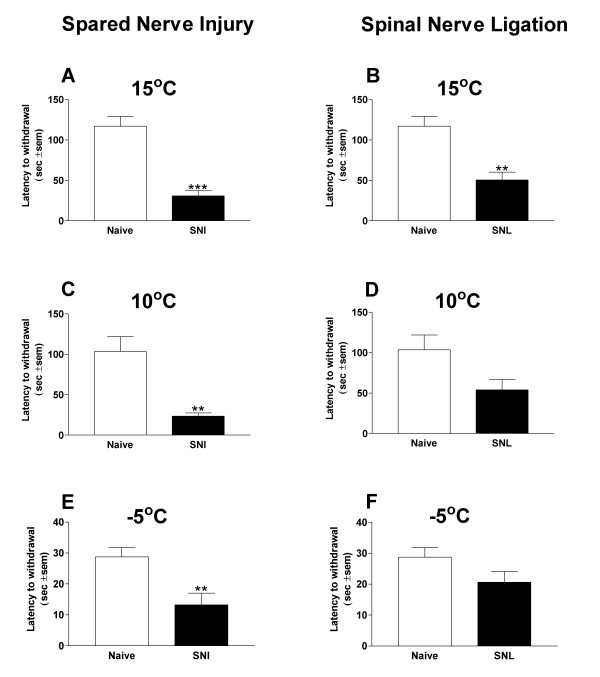
Cold allodynia and cold hyperalgesia in the Spared Nerve Injury and Spinal Nerve Ligation models of neuropathic pain. At 15°C both the SNI and SNL have significantly reduced latencies to withdrawal (A and B) compared to both naïve controls (n = 6). At 10°C the SNI model shows a significantly reduced latency (C), but the SNL model does not (D). The SNI shows cold allodynia (a significantly reduced response to a non-noxious temperature) at both 10°C and 15°C. The SNL model only displays this at 15°C. At -5°C, a temperature known to be painful in the naïve rat, the SNI model displays a further reduction in withdrawal latency (E); the SNL does not show any further reduction (F). Data are mean ± S.E.M.

Two to four weeks after SNI or SNL surgery, the treated rats and naïve controls were tested at cold plate surface temperatures of: 15°C, 10°C and -5°C (Fig 2)

### Spared nerve injury

At 15°C and 10°C the withdrawal latency was significantly reduced compared to naïve controls (Figs 2A and 2C) (p < 0.001 and p < 0.01 respectively). Since these temperatures did not elicit a reduced withdrawal response in naïve animals, we ascribe the presence of such a response in the SNI rats as a manifestation of cold allodynia. At -5°C, a temperature that elicits a large reduction in withdrawal response in naïve rats, the withdrawal latency of the SNI animals was even lower than naïve animals (p < 0.01) (Fig 2E). We conclude that this accelerated response reflects cold hyperalgesia.

### Spinal nerve ligation

The SNL model of neuropathic pain differs from the SNI model. At 15°C SNL rats showed significantly reduced latencies (p < 0.01) (Fig 2B), at 10°C the latency is reduced, but not significantly (Fig 2D), and at -5°C there was no difference between SNL and naïve withdrawal thresholds (Fig 2F). Therefore, the SNL model shows cold allodynia but not cold hyperalgesia at the temperatures tested.

### Temporal development of cold allodynia in the SNI

**Figure 3:**
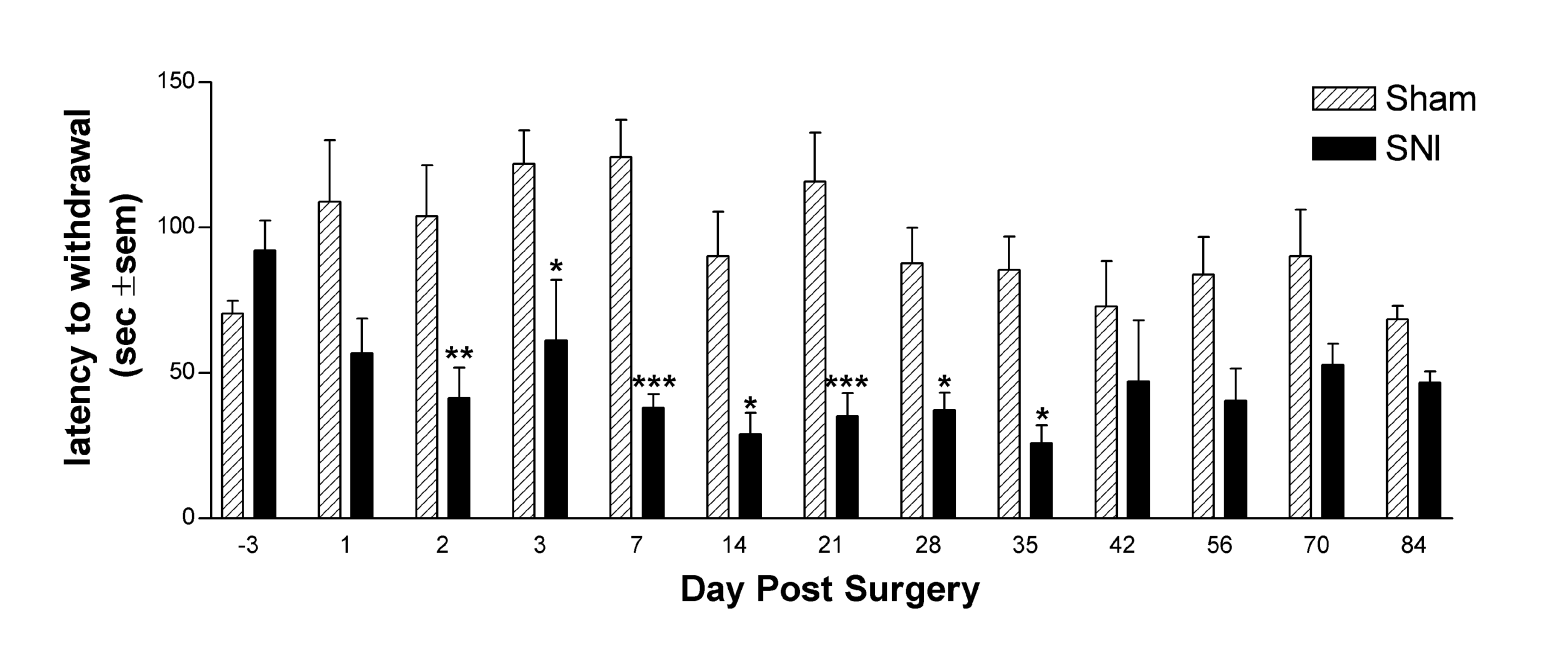
Temporal development of cold allodynia (cold plate surface temperature 10°C) in the Spared Nerve Injury model of neuropathic pain. Following SNI (n = 6), the latency to withdrawal is consistently lower than sham controls (n = 6). It is significantly lower from 2 days to 5 weeks post surgery. Data are mean ± S.E.M.

We examined the development of cold allodynia at 10°C in SNI rats over time (Fig 3) and found a reduction in withdrawal latency from day one to 12 weeks post surgery. This reduction was significantly different from sham controls at all time points from 2 days to 5 weeks post surgery.

### CFA inflammation of the hind paw

**Figure 4:**
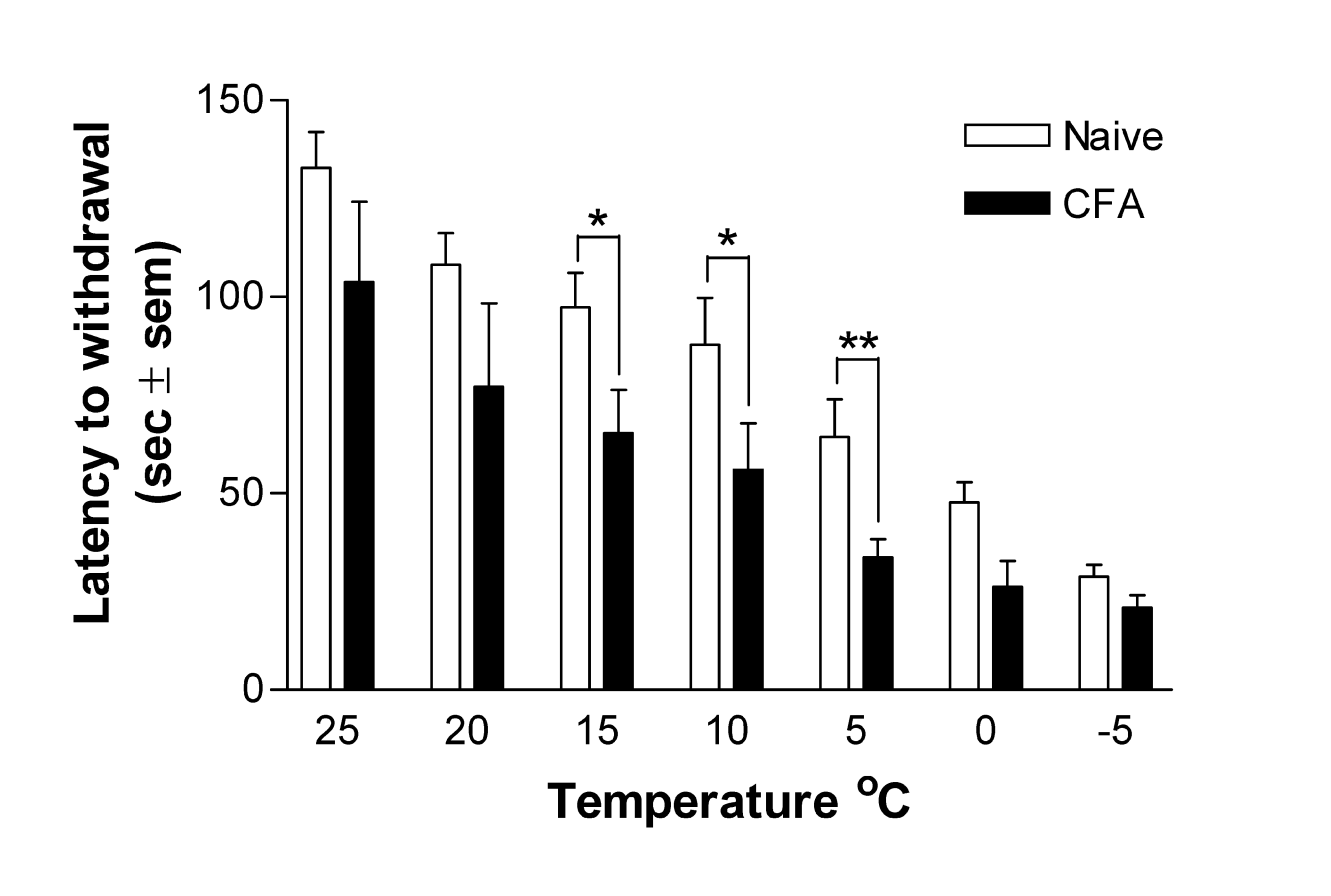
Cold sensitivity following CFA inflammation of the hindpaw. The latency to withdrawal following intraplantar injection of CFA (n = 6 to 12) is significantly lower than naïve controls (n = 6 to 12) at 15°C, 10°C and 5°C. From this we can conclude that CFA induced inflammation of the hindpaw has both cold allodynic and cold hyperalgesic components. Data are mean ± S.E.M.

Inflammation was induced in one hindpaw by an intraplantar injection of CFA. Responses were tested over a full range of temperatures (-5°C to 25°C, at 5°C intervals) and compared to naïve controls (Fig 4). The latency to withdrawal was consistently lower in the CFA treated rats than the naïve controls at all temperatures tested, and was significantly different from naïve animals at 15°C (p < 0.05), 10°C (p < 0.01) and 5°C (p < 0.01). Therefore, CFA-induced peripheral inflammation may elicit both cold allodynia and cold hyperalgesia.

## Discussion

A Peltier cooled cold plate allows accurate assessment of cold pain and cold allodynia thresholds in unrestrained rats. Measuring the latency to the first hindpaw withdrawal rather than cumulative responses to a fixed time ensures that the animals only have a short exposure to the cold surface. This is likely to be important at low temperatures (below 0°C) where the skin may be damaged by the cold. We find, moreover, that detection of the time to a brisk lift or stamp of the hindpaw is easier than counting total paw-lifts over a longer period of time. In naïve adult male Sprague-Dawley rats the latency to withdrawal, when measured using this apparatus, decreases as the cold plate surface temperature is lowered, and is significantly different from the ambient temperature baseline at temperatures of 5°C and below. From these observations we conclude that the temperature threshold for eliciting cold pain in these animals is in the range of 5–9°C and that temperatures of 10°C or higher are innocuous.

Cold allodynia, an increased sensitivity to normally non-painful cool temperatures, is a characteristic feature of clinical neuropathic pain states. One clinical study has shown that in normal control subjects the cold pain detection threshold is 8.4°C, compared to one of 23.8°C in those subjects suffering from post herpetic or post-traumatic neuralgia [19]. In our experiments, both the SNI and SNL rat models of neuropathic pain result in increased sensitivity to a normally innocuous (15°C) stimulus. Cold allodynia in the SNI model is present from 2 to 35 days post surgery.

When the testing temperature is reduced to -5°C (a painful temperature for naïve rats) SNI rats show an even lower latency of response. This could represent cold hyperalgesia, an increased sensitivity to an already painful cold stimulus. Interestingly we did not detect cold hyperalgesia in the SNL model at -5°C, even though an increase in the percentage of cold responsive neurons in the uninjured L4 DRG following SNL has been reported [20].

In rats with an inflamed hindpaw, the latency to paw withdrawal upon exposure to the cold plate was consistently lower than naïve control animals over the full range of temperatures tested. This is perhaps surprising as cold is commonly used to treat minor traumatic injuries. However, persistent exposure to cold is likely to anesthetize or desensitize the skin and thus may mask or override any initial cold allodynia. Earlier studies have shown an increased sensitivity to cool stimuli following CFA-induced inflammation [7,21]. We find significantly reduced responses at 15°C and 10°C, two normally innocuous temperatures in naïve rats and a shortened latency at 5°C, a painful temperature in naïve animals. These observations lead us to conclude that peripheral inflammation may have both allodynic and hyperalgesic components. This occurs in spite of an elevation in foot surface temperature of between 2°C and 7°C [22]. In contrast, there is no difference in plantar surface temperature between the ipsi-lateral and contra-lateral paws following SNL [23].

TRPM8 and TRPA1 are two members of the transient receptor potential superfamily of nociceptor transduction ion channels with cool or cold activation temperatures. In heterologous expression systems TRPM8 has an activation temperature of less than 23°C,[16,17] and TRPA1 an activation temperature of less then 18°C [18], although not all studies report cold sensitivity for this channel [24,25]. It is however difficult to relate these activation temperatures to temperature stimuli applied to skin *in vivo*. A cool stimulus in contact with the skin will produce a temperature gradient between the skin surface and the underlying tissue that will, over time, cool nociceptor terminals in the epidermis to a point at which the cool transduction channels are activated. This will depend on the external temperature, skin temperature, body temperature, skin thickness and blood flow as well as the activation threshold of the ion channel. In order to elicit a response, a generator potential needs to be produced in nociceptor peripheral terminals that is sufficiently large to initiate conduction of action potentials at high enough frequency and in enough afferents to activate a response in the CNS. The size of the generator potential will reflect the temperature, the numbers of cool sensitive ion channels and membrane excitability. The numbers of afferents activated will depend on the surface exposed to the cold stimulus, the temperature and the threshold of nociceptors.

## Conclusion

The threshold for eliciting a cold pain withdrawal response in naïve Sprague-Dawley rats is 5–9°C. The SNI and SNL models of neuropathic pain both show increased sensitivity at 15°C, a non-painful temperature in the naïve rat, this we interpreted as cold allodynia. The SNI model also showed increased sensitivity at -5°C, this we interpret as a cold hyperalgesic response. The CFA model of peripheral inflammation also exhibited cold allodynic components at 10°C and 15°C and hyperalgesic components at 5°C.

We feel that novel analgesics designed for the treatment of pain should include tests for activity against cold allodynia and hyperalgesia.

## Methods

**Figure 5:**
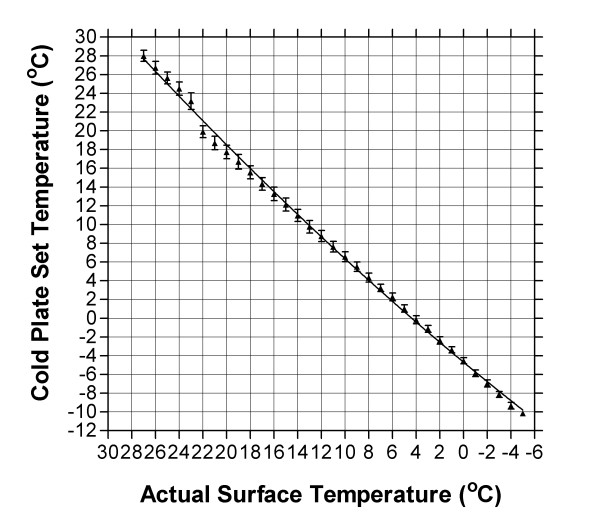
The relationship between cold plate set temperature (y-axis) and actual surface temperature (x-axis). Surface temperatures were measured at 4 different positions on the cold plate and mean ± S.E.M. temperature obtained. The discrepancy between set and actual temperature is due to the cold plate temperature probe being positioned in the core of the plate rather than on the surface. All temperatures used in this study are surface temperature.

The Peltier effect occurs when an electrical current is passed through two dissimilar metals or semi-conductors (n-type and p-type) connected at Peltier junctions. The current drives a transfer of heat from one junction to the other causing one junction to cool off while the other heats up. Thus the surface temperature of a cold plate can be controlled and adjusted by varying current. A commercially available Peltier cooled cold plate, including a temperature controller and heat sink (TECA Chicago, Il) was equipped with a Plexiglas box (18 cm × 14 cm × 14 cm) to contain test animals. First, using a temperature-measuring probe (SPER Scientific, Scottsdale Az), the surface temperature of the plate was thoroughly assessed over a full range of temperatures (-5°C to 25°C) to assess if the controller temperature shown was indeed surface temperature. This proved not to be the case as the probe monitoring the temperature of the cold plate is positioned such that it records the "core" temperature of the cold plate and not its surface temperature. We therefore created a conversion graph (Fig 5). All temperatures stated in this study are cold plate surface temperature obtained using this conversion graph.

### Animals

Male Sprague-Dawley rats (Charles River Lab, Wilmington, MA), initially weighing 175–200 g, were housed in cages with a thick sawdust bedding, had free access to water and food and were exposed to a standard 12:12 hour on/off light cycle. Experiments were approved by the Animal Care Committee of the Massachusetts General Hospital according to the ethical guidelines of the International Association for the Study of Pain [26]. Following surgery animals were allowed to recover fully from anesthesia before being returned to their home cage.

### Neuropathic pain model surgery

#### Spared nerve injury (SNI)

Under isoflurane anesthesia (3% induction, 1.5% maintenance), the left sciatic nerve was exposed at mid-thigh level, and the tibial, common peroneal and sural nerves identified. The tibial and common peroneal nerves were ligated with 5/0 silk and cut, taking care to avoid any lesion to, or stretching of the intact sural nerve [19]. Muscle and skin were then closed in two layers.

#### Spinal nerve ligation (SNL)

Under isoflurane anesthesia (3% induction, 1.5% maintenance) the L5 spinal nerve was exposed distal to the L5 dorsal root ganglion. It was then tightly ligated using 7/0 silk and the muscle and skin closed in two layers [18].

### Induction of inflammation

Complete Freunds Adjuvant (CFA) inflammation: Under isoflurane anesthesia (3% induction, 1.5% maintenance), 100 μl of CFA (Sigma) was injected subcutaneously into the center of the plantar surface of the left hindpaw using a 27 gauge needle.

### Behavioral testing

Two protocols were employed: Temperature effect; testing the models over a range of temperatures, and temporal development; testing a given model (SNI) at one defined temperature over a time course of 12 weeks.

### Temperature effect experiments

For neuropathic pain temperature effect experiments, rats were allowed to recover from surgery for 2 weeks before testing. These animals were then tested a maximum of twice per week. The temperature for each test was selected at random to reduce the possibility of a learning type response. In the inflammatory experiment, rats were tested twice, once 48 hours following CFA injection and again 24 hours later, to ensure that testing was carried out when the inflammatory response was at its peak.

### Temporal development of cold allodynia experiments

Rats were baseline tested twice prior to SNI or sham surgery. Following surgery, rats were tested on days 1, 2, 3 and 7 and then once every 7 days for 12 weeks.

### Testing protocol

The temperature of the cold plate was set and allowed to stabilize for 5 minutes (ambient temperature of testing room 21°C ± 1°C). The animal was then placed onto the cold plate and the time taken for the first brisk lift or stamp of the ipsilateral hindpaw to occur was recorded. Locomotor movements were quite distinct, involving coordinate movement of all four limbs, and these were excluded. We interpret the time to the brisk response as the latency for cold pain withdrawal. Given that temperatures of 15°C and above are considered based on human psychophysical data, innocuous, and that rats explore their environment, we chose 100 seconds as the upper time limit for a cold "pain" response. Any response with a latency greater than 100 seconds was considered non painful. A maximum cut off time of 150 seconds was used to prevent tissue damage at the lower temperatures. Each rat was only tested once on any given test day to avoid any possible anesthetic or tissue damage effects that could be produced by repeated exposure to a cold surface. All experiments were carried out in groups of 5 to 10 experimental animals and an equivalent number of sham or naïve controls, with the tester blinded to the treatment

### Statistical analyses

Results are presented as mean ± standard error of the mean (SEM). Data were analyzed using Prism software (Graphpad Inc., San Diego, CA). Naïve temperature effect data was analyzed using a one-way ANOVA with Dunnetts multiple comparison post-test. Neuropathic and inflammatory temperature effect data were analyzed using a one-way ANOVA with Newman-Keuls multiple comparison post-test. Temporal development was analyzed using a two-way ANOVA with Bonferroni post-tests. A P-value of < 0.05 (*), P < 0.01 (**) and P < 0.001 (***) were chosen as indicating significance.

## Authors' contributions

AJA and DCB conceived, designed and carried out the study. CJW conceived and coordinated the study.
